# INDIVIDUAL AND COUNTY-LEVEL FACTORS OF SERVICE USE BY DEMENTIA FAMILY CAREGIVERS IN RURAL APPALACHIA

**DOI:** 10.1093/geroni/igad104.1496

**Published:** 2023-12-21

**Authors:** Suyoung Nah, Tina Savla, Karen Roberto

**Affiliations:** Virginia Polytechnic Institute and State University, Blacksburg, Virginia, United States; Virginia Tech, Blacksburg, Virginia, United States; Virginia Polytechnic Institute & State University, Blacksburg, Virginia, United States

## Abstract

Formal services have been shown to provide health benefits for care recipients and family caregivers. Despite prior efforts, it is still unclear which factors play a role in the decisions to use formal services by family caregivers living in under-resourced rural regions. Drawing upon the Andersen model of health service use, this study examined individual and county-level factors’ associations with the utilization of support services (e.g., homemaker, respite) and personal services (e.g., home health nurse, personal care aide) by dementia family caregivers residing in rural Appalachia (N = 163). Results of multilevel models revealed that caregivers with higher income and care recipients with more limitations in activities of daily living tended to use more support and personal services. Greater informal support from family or friends was associated with less utilization of support services, but not personal services. Caregivers residing in counties with higher poverty and lower public spending on support services for older adults were more likely to use support services. Conversely, caregivers living in counties with more home health agencies were more likely to utilize personal services. These findings suggest that caregivers living in rural regions may depend more on informal support for meeting basic care needs of their relative living with dementia. However, if the care recipient requires more specialized care, living in resource-rich counties increases their chances of receiving appropriate care. Discussion will focus on strategies to allocate funds and resources to expand care coverage to vulnerable individuals such as people living with dementia in rural regions.

